# Valuing end-of-life care: translation and content validation of the ICECAP-SCM measure

**DOI:** 10.1186/s12904-021-00722-5

**Published:** 2021-02-08

**Authors:** Uta Gühne, Marie Dorow, Jessica Grothe, Janine Stein, Margrit Löbner, Judith Dams, Joanna Coast, Philip Kinghorn, Hans-Helmut König, Steffi G. Riedel-Heller

**Affiliations:** 1 Institute of Social Medicine, Occupational Health and Public Health (ISAP) Medical Faculty, University of Leipzig, Philipp-Rosenthal-Str. 55, 04103 Leipzig, Germany; 2 Department of Health Economics and Health Services Research, University Medical Centre Hamburg-Eppendorf (UKE), Hamburg, Germany; 3 Health Economics Bristol, Population Health Sciences, Bristol Medical School, University of Bristol, Bristol, UK; 4 Health Economics Unit, Institute of Applied Health Research, University of Birmingham, Edgbaston, UK

**Keywords:** Palliative care, Quality of life, Hospices, Translations, Questionnaires

## Abstract

**Background:**

The evaluation of care strategies at the end of life is particularly important due to the globally increasing proportion of very old people in need of care. The ICECAP-Supportive Care Measure is a self-complete questionnaire developed in the UK to evaluate palliative and supportive care by measuring patient’s wellbeing in terms of ‘capability’. It is a new measure with high potential for broad and international use. The aims of this study were the translation of the ICECAP-Supportive Care Measure from English into German and the content validation of this version.

**Methods:**

A multi-step and team-based translation process based on the TRAPD model was performed. An expert survey was carried out to assess content validity. The expert panel (*n* = 20) consisted of four expert groups: representative seniors aged 65+, patients aged 65+ living in residential care, patients aged 65+ receiving end-of-life care, and professionals in end-of-life care.

**Results:**

The German version of the ICECAP-Supportive Care Measure showed an excellent content validity on both item- and scale-level. In addition, a high agreement regarding the length of the single items and the total length of the questionnaire as well as the number of answer categories was reached.

**Conclusions:**

The German ICECAP-SCM is a valid tool to assess the quality of life at the end of life that is suitable for use in different settings. The questionnaire may be utilized in multinational clinical and economic evaluations of end-of-life care.

**Supplementary Information:**

The online version contains supplementary material available at 10.1186/s12904-021-00722-5.

## Background

Due to demographic changes, end-of-life care has developed in importance and represents a growing socio-political challenge [1–3]. The steady increase in the older population is accompanied by growing multi-morbidity, rising health care needs and higher medical expenses [4–7]. In this context, it is necessary to provide valid measures to assess the quality of life for those at the end of life. Quality of life is an important outcome variable in evaluating health care strategies and determining the cost-effectiveness of health care interventions. Current measures of quality of life in older people focus mainly on health and physical functioning, neglecting domains that may be of particular relevance for the oldest old [8]. Likewise, prior research on preferences for end-of-life care in the elderly has mainly concentrated on treatment decisions including life-sustaining methods [9]. To address this research gap, Sutton and Coast conducted a qualitative study [10], that identified attributes that older people consider important for a ‘good death’ and developed a supportive care measure (ICECAP-SCM) based on their findings. The ICECAP-SCM contains the following seven attributes: *choice*, *love and affection, freedom from physical suffering and emotional suffering*, *dignity*, *being supported* and *preparation*. The ICECAP-SCM is based on Sen’s capability approach [11] which focuses on wellbeing in terms of an individual’s capabilities, that is what they are able to do and be in their lives [12].

Research using the ICECAP-SCM to measure capability at the end of life has highlighted its feasibility for use in hospice settings, where it was preferred over other measures of quality of life, particularly for those coming towards the very end of their lives [13]. A particular benefit of the ICECAP-SCM is the work that has been done on generating meaningful values, representing the extent of change in a person’s capability that arises from changes in the different attributes of the measure. Such valuation work has been largely conducted in the general population [14], but preliminary work has also been conducted with those at the end of life [15].

Even though international studies [16–19] have shown that a good death is influenced by attributes beyond physical health, there is a lack of validated questionnaires to assess quality of life at the end of life in German speaking countries. Therefore, the aims of the study were (1) the translation of the ICECAP-SCM into German, and (2) the content validation of the German version of the ICECAP-SCM using an expert panel. The overarching goal was to provide a measure for the assessment of quality of life at the end of life that may serve as a base to enhance the effectiveness and cost-effectiveness of palliative care strategies.

## Methods

### Ethics approval

The study was approved by the Ethics Committee of the University of Leipzig (ref: 503/17-ek; date of approval: 24 January 2018).

### Translation process and study design

**Fig. 1:**
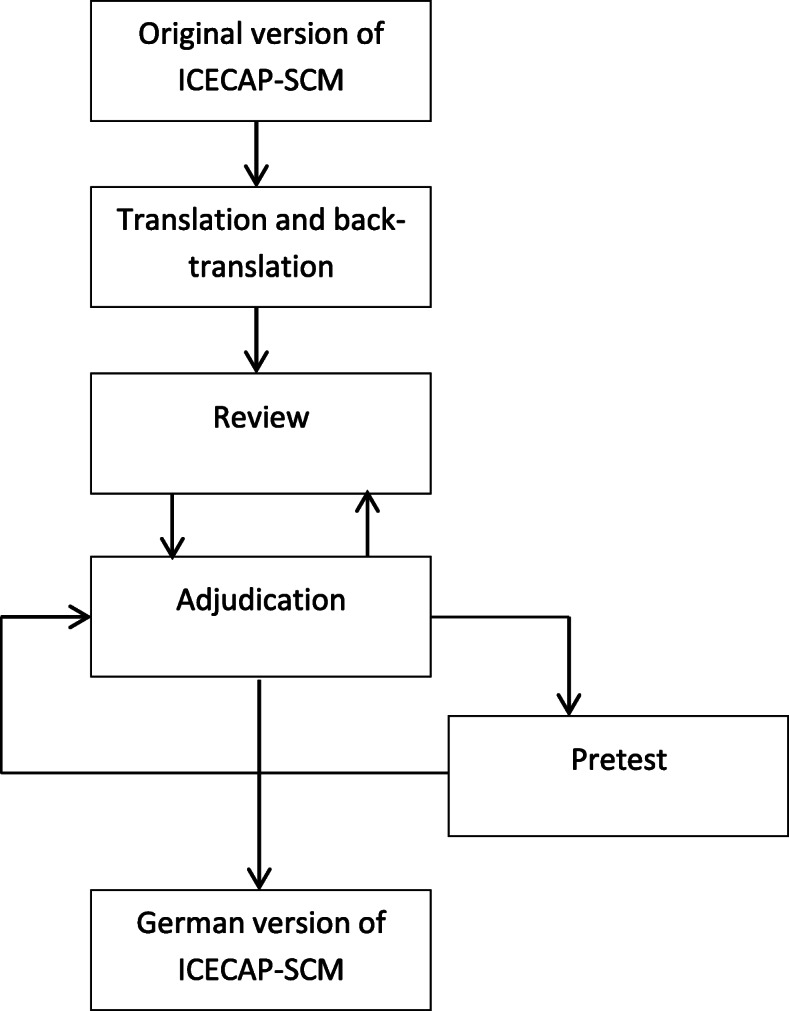
Translation of ICECAP-SCM based on the TRAPD-model (according to Harkness [20])

We performed a multi-step and team-based translation process based on the **TRAPD** model (**T**ranslation, **R**eview, **A**djudication, **P**retesting, and **D**ocumentation; see Fig. 1). In this case, several people are involved in the translation and the process is carried out in several stages. The probability of incorrect translations can thus be significantly reduced [21]. The team approach is complemented by pre-test procedures and careful process documentation. TRAPD was originally developed by Janet Harkness and is described in the guidelines of the “European Social Survey Programme” or in the “Cross-Cultural Survey Guidelines” for the translation and adaption of questionnaires [20, 22]. Initially, the original English version of the ICECAP-SCM was translated into German by two independent translators. Subsequently, both translators discussed their translations together with other independent persons. The first draft of the German ICECAP-SCM was then translated back into English by another translator. The back-translation was discussed with the original developers and final adjustments were undertaken resulting in a preliminary German version of the ICECAP-SCM. The translation process lasted from beginning of November 2017 until end of January 2018.

As part of the pretesting, we aimed to evaluate the content validity of this preliminary version and to investigate if further changes of the questionnaire were necessary in order to provide a valid instrument that could be applied in end-of-life care in a German-speaking environment. Therefore, an expert survey with 20 participants was conducted following the Delphi technique.

### Sampling and recruitment

The expert panel (*n* = 20) for content validation of the ICECAP-SCM consisted of four expert groups: *n* = 5 representative seniors aged 65+ within the general population, *n* = 5 patients aged 65+ living in residential care, *n* = 5 patients aged 65+ receiving end-of-life care, and *n* = 5 professionals in end-of-life care. The first three of these groups mirrored the groups included in the development of the original ICECAP-SCM [10]. Patients were excluded if they had moderate or severe dementia. All participants had to have sufficient knowledge of the German language. The participants were recruited via existing networks within the Institute for Social Medicine, Occupational Health and Public Health (ISAP), i.e. patients in residential care were recruited via a cooperating general practitioner, while patients and professionals in end-of-life care were recruited via the palliative care unit of the University of Leipzig. Study participants from the general population and professionals in end-of-life care were informed about the study via an information sheet and were asked to provide written informed consent to participate in the survey. Next, they received a structured questionnaire via post and were asked to fill it in and send it back to the ISAP. Patients in residential care and end-of-life care received both oral and written information about the purpose of the study and were asked to give written informed consent to participate. These patients were interviewed face-to-face by trained interviewers using the same structured questionnaire as the general population and professionals in end-of-life care. All experts received a monetary incentive of 80 euros for their participation.

### Data collection

Data collection within the expert survey lasted from beginning of February until end of June 2018. The structured questionnaire presented to the experts included sociodemographic data and both closed and open questions concerning the ICECAP-SCM.

Sociodemographic data included the participants’ age, gender, level of school education (no school graduation, still in school, general elementary education, intermediate general qualification/general maturity, polytechnic secondary, advanced technical college certificate/Abitur or high school graduation, other), level of professional education (no vocational education, still in education, vocational and occupational/school apprenticeship, specialists/technicians or master school, technical degree or high school degree, postgraduate degree or doctorate, other) and, for professionals in end-of-life care, vocational qualification and years of work experience. Furthermore, all experts were asked if they were familiar with the subject ‘health care and quality of life at the end of life’ (not familiar at all, familiar, and very familiar).

In order to evaluate the content validity, participants were asked questions about the relevance and clarity of items presented in ICECAP-SCM, the integrity, structure and length of the questionnaire with regard to its applicability in palliative care. To examine the relevance of items, experts were asked with a 4-point-Likert scale if each of the seven domains was relevant for the assessment of quality of life in end-of-life care (1 = not relevant, 2 = somewhat relevant, 3 = quite relevant, 4 = highly relevant). Likewise, participants were asked whether each of the seven domains was formulated in a clear and understandable way (1 = item is not clear, 2 = item needs moderate changes, 3 = item needs minor changes, 4 = item is clear). Experts had the possibility to make suggestions for improvement for each domain. Furthermore, experts were asked if, in their view, any further domains should be added to the ICECAP-SCM (yes/no; free comments) and if any domains were redundant (yes/no; free comments). Regarding length and structure of the ICECAP-SCM, participants were asked whether the length of the total questionnaire, the length of the single items as well as the number of four answer categories per domain was appropriate for applicability in end-of-life care (yes/no; free comments). At the end of the questionnaire, they were given the chance to make general comments or suggestions about the questionnaire. Table 1 gives an overview of the ICECAP-SCM domains. Each domain contains exactly one question and four corresponding answer options. The original questionnaire thus contains a total of 7 questions (see Additional file 1).

**Table 1:** ICECAP-SCM domains

ICECAP-SCM domain	Formulation in ICECAP-SCM
Choice	Having a say: Your ability to influence where you would like to live or be cared for, the kind of treatment you receive, the people who care for you.
Love and affection	Being with people who care about you: Being with family, friends or caring professionals.
Physical suffering	Physical suffering: Experiencing pain or physical discomfort which interferes with your daily activities
Emotional suffering	Emotional suffering: Experiencing worry or distress, feeling like a burden.
Dignity and self-respect	Dignity: Being treated with respect, being spoken to with respect, having your religious or spiritual beliefs respected, being able to be yourself, being clean, having privacy.
Support	Being supported: Having help and support.
Preparation	Being prepared: Having financial affairs in order, having your funeral planned, saying goodbye to family and friends, resolving things that are important to you, having treatment preferences in writing or making a living will.

There is the possibility to tick one of 4 attributes in each domain, i. e. always (4), often (3), sometimes (2), rarely (1) or most of the time (4), some of the time (3), only a little of the time (2), never (1). Thereby it will be checked to what extent the respective statement applies to the current situation

### Data analysis

A descriptive data analysis was performed using the statistic software programs Stata 13 (StataCorp 2013. Stata Statistical Software: Release 13. College Station, TX: StataCorp LP) and IBM SPSS Statistics for Windows, version 24 (IBM Corp., Armonk. NY). In addition, experts’ comments were considered for the final validated version of the German ICECAP-SCM.

The quantitative analysis of the relevance and clarity was calculated using Content Validity Indices (CVIs) [23, 24]. The CVI is an established index for the content validity of scales [25]. To determine item-specific CVIs (I-CVIs) for each of the seven domains of the ICECAP-SCM, the answer categories for relevance and clarity were dichotomized for each item. For relevance, answer categories 1 and 2 were combined to category 1 (“not relevant”) and categories 3 and 4 were combined to category 2 (“relevant”). For clarity, answer categories 1 and 2 were combined to category 1 (“not clear”) and categories 3 and 4 were combined to category 2 (“clear”). The I-CVIs for relevance (clarity) were computed as the number of experts giving the rating relevant (clear) for an item, divided by the number of all experts. In addition, two Scale Content Validity Indices (S-CVIs) were calculated to interpret the overall relevance and clarity of ICECAP-SCM. The S-CVI is defined as the average I-CVI (S-CVI/Ave) across items [25]. Polit et al. recommend a S-CVI of at least 0.90 and I-CVIs of 0.78 or higher for a scale to be judged as having excellent content validity [25].

## Results

### Sample characteristics

Table 2 shows the sample characteristics across expert groups. The vast majority was female (*n* = 18; 90%) and the mean age was 68.6 years (SD = 12.7). Half of the participants completed polytechnic secondary school (*n* = 10; 50%), *n* = 9 (45%) had an advanced technical college or high school degree and n = 1 (5%) finished general elementary school. Five participants (25%) completed an apprenticeship and another five participants (25%) had a master school degree. Four participants (20%) had a doctorate and *n* = 3 (15%) had a university degree. Three participants (15%) had no vocational qualification or were still undergoing vocational training. The majority (*n* = 13; 65%) was familiar with the topic “health care and quality of life at the end of life”. Professionals in end-of-life care had a mean work experience of 10 years (SD = 1.87). This subgroup consisted of *n* = 1 social worker, *n* = 1 specialist nurse, *n* = 1 physiotherapist and psychological counsellor, *n* = 1 psychological psychotherapist and *n* = 1 physician.

**Table 2:** Sample characteristics

	General population (***n*** = 5)	Residential care (***n*** = 5)	End-of-life care patients (***n*** = 5)	End-of-life care professionals (***n*** = 5)	Total (***n*** = 20)
**Female (n (%))**	4 (80)	4 (80)	5 (100)	5 (100)	18 (90)
**Age (M** ***(SD)*)**	77 (3.67)	77.6 (9.45)	69.4 (3.85)	50.4 (6.66)	68.6 (12.7)
**Education (n (%))**					
General elementary education	0	0	1 (20)	0	1 (5)
Polytechnic secondary school	2 (40)	5 (100)	2 (40)	1 (20)	10 (50)
Advanced technical college certificate/Abitur or high school	3 (60)	0	2 (40)	4 (80)	9 (45)
**Level of professional education (n (%))**					
None	0	2 (40)	0	0	2 (10)
Still in education	0	0	1 (20)	0	1 (5)
Vocational and occupational/ school apprenticeship	2 (40)	2 (40)	1 (20)	0	5 (25)
Specialists/ technicians or master school	2 (40)	1 (20)	1 (20)	1 (20)	5 (25)
Polytechnic degree or university degree	0	0	1 (20)	2 (40)	3 (15)
Postgraduate degree or doctorate	1 (20)	0	1 (20)	2 (40)	4 (20)
**Familiarity with ‘health care and quality of life at the end of life’ (n (%))**					
Not familiar at all	0	0	2 (40)	0	2 (10)
Familiar	5 (100)	5 (100)	3 (60)	0	13 (65)
Very familiar	0	0	0	5 (100)	5 (25)

*M* mean, *SD* standard deviation

### Content validity

Table 3 shows the CVIs for the seven domains of ICECAP-SCM concerning relevance and clarity. The I-CVIs for relevance ranged between 0.85 (domain preparation) and 1 (domains love and affection, physical suffering, dignity and self-respect, support) indicating excellent content validity on both item- and scale-level (S-CVI/Ave = 0.96). Likewise, item- and scale-level content validity for clarity were excellent since all I-CVIs reached the maximum value of 1.

**Table 3:** Expert ratings on relevance and clarity of ICECAP-SCM domains

	Relevance			Clarity		
ICECAP-SCM domain	A_**R**_	I-CVI	Evaluation	A_**C**_	I-CVI	Evaluation
Choice	19	0.95	Excellent	20	1	Excellent
Love and affection	20	1	Excellent	20	1	Excellent
Physical suffering	20	1	Excellent	20	1	Excellent
Emotional suffering	19	0.95	Excellent	20	1	Excellent
Dignity and self-respect	20	1	Excellent	20	1	Excellent
Support	20	1	Excellent	20	1	Excellent
Preparation	17	0.85	Excellent	20	1	Excellent
**S-CVI/Ave**		0.96	Excellent		1	Excellent

*A*_*R*_ number of experts giving a rating of either 3 or 4 on relevance, *A*_*C*_ number of experts giving a rating of either 3 or 4 on clarity, *I-CVI* item-level content validity index, *S-CVI/Ave* scale-level content validity index, averaging method

### Improvement suggestions

All experts (*n* = 20; 100%) agreed that the length of the single items was appropriate for applicability in end-of-life care. Moreover, 19 out of 20 experts (95%) stated that the total length of the questionnaire was adequate for use in palliative care. Likewise, most experts approved the number of four answer categories (*n* = 18; 90%). No ICECAP-SCM domain was judged as being redundant by any expert. In contrast, 15 out of 20 participants (75%) stated that no further domains should be added to the ICECAP-SCM. Three experts from the general public, one patient and one professional in palliative care suggested that further domains should be added. Those were inner peace, being socially and politically involved or informed, public transport and accessibility, support from relatives, financial situation, social contact, tasks/being needed, vitality, sociability, diversion and humour. Due to the heterogeneity of the reported domains and the extent to which some (e. g. support from relatives, social contact) related to existing attributes, we added the following question and a free text box to the German version of the ICECAP-SCM: “Are there any other aspects that are important for your current well-being?” (yes/no; if yes, please specify).

## Discussion

The ICECAP-SCM is a self-complete questionnaire developed to evaluate palliative and supportive care by measuring patients’ wellbeing in terms of ‘capability’. Since the ICECAP-SCM was developed in English, this study aimed to provide a German version of the ICECAP-SCM questionnaire, and present early evidence of validity for use in research and clinics in German-speaking countries. To provide equivalence between the English and the German versions of the ICECAP-SCM, a multi-step and team-based translation process based on the TRAPD model [20] was followed. The content validity was evaluated using an expert survey and the calculation of CVIs [23, 25, 26].

The findings of this study highlight the excellent content validity of the German version of ICECAP-SCM. Due to the consistent positive ratings on relevance and clarity of the presented domains, no changes were made on the item level. A disadvantage of using the CVIs is the lack of adjustment for a chance agreement [27]. Therefore, multi-rater kappa statistics are additionally recommended as an index indicating the degree of agreement beyond chance. Although Polit et al. postulate that the calculation of multi-rater kappa statistic is hardly necessary if the number of experts is 10 or more [25], we additionally calculated the k* index within each dimension. The results did not lead to any additional information or quality gain. Based on the qualitative evaluation of free comments, an additional open question was added to the German version of ICECAP-SCM. This way, patients who will be interviewed with the German ICECAP-SCM have the chance to name individual domains that are of particular relevance for their quality of life. Although this cannot contribute to the overall score arising from the ICECAP-SCM for use in economic evaluations, it does provide additional information for use in the research more generally.

The results of this study are consistent with the results of previous research. Using discrete choice experiments and best-worst scaling tasks, Coast et al. were able to show that the four-step response scale is most appropriate [28]. In line with these findings, the current study showed that the number of four response levels is appropriate for use with older people at the end of life. This research also showed that the items seemed meaningful and easy to complete in line with UK hospice-based research [13]. Kinghorn and Coast were able to confirm the relevance of the seven ICECAP-SCM domains based on a qualitative survey of experts [29]. The current results also point to practicability by relevant target persons.

The systematic translation process with content validation of the instrument opens research for transnational comparisons and data pooling [20]. To include different perspectives, the individuals involved in this study consisted of a broad expert panel including providers who have expertise in end-of-life care and lay experts with different backgrounds [30] that mirrored those included in the original development of the ICECAP-SCM [10]. Suggestions made within the scientific literature regarding the number of experts required differs and ranges from 3 to 20 experts [23, 30]. In the present work a total of 20 experts were interviewed.

The present study has some limitations. First, a limitation of the results is linked to the fact that almost all respondents were female. A second limitation may be the fact that participants from the general population and professionals in end-of-life care filled a structured questionnaire which they received by post while patients in residential care and end-of-life care were interviewed face-to-face. While both survey modes have their qualification, it is recommended to use only one mode within a study as different strengths and weaknesses of the procedures entail different effects that can influence the results [31]. Here, a balanced result was achieved despite the mix of data collection methods. Criteria such as costs and response rates are unlikely to be relevant for a number of 20 respondents. However, interactions between interviewers and respondents in personal interviews are strong and influences are difficult to control; nevertheless, the interviewers in our study were trained to counter such effects. Completing a written questionnaire might not always be feasible for participants, as it was the case for the people personally interviewed in this study.

Providing valid measures for the assessment of quality of life at the end of life in different languages is a crucial step to improving palliative care in ageing societies. This study provides a German version of ICECAP-SCM that is publicly available (https://www.birmingham.ac.uk/Documents/college-mds/applied-health/health-informatics/ICECAP-validiert-German.pdf) and at the same time gives early evidence on content validity. The German ICECAP-SCM is an innovative tool to assess capability and sense of wellbeing at the end of life that is suitable for use in hospices, palliative care units and nursing homes. Our project partners in the Department of Health Economics and Health Services Research at the University Medical Center Hamburg-Eppendorf (UKE) are currently conducting a large representative survey of the German general population. This study aims to determine preference-based value sets (tariffs) for the ICECAP-SCM based on preferences of the German general population. Thus, the German ICECAP-SCM can be used as effectiveness measure in health economic analyses in addition to health-related quality of life. In a clinical context, the ICECAP-SCM may help to establish and improve health care interventions that reflect the particular needs of individuals at the end of life. Furthermore, the ICECAP-SCM may serve to detect target groups in the older population with higher treatment demands. Future studies should identify modifiable and setting specific factors associated with quality of life at the end of life to enhance need-based palliative care strategies.

## Conclusions

The assessment of care strategies at the end of life is particularly important due to the globally increasing proportion of older and very old people in need of care. Currently, data on the health-related quality of life of older palliative care patients are scarce, as well-evaluated instruments for measuring quality of life or for health economic analyses are not internationally available. This study provides a validated German version of the ICECAP-SCM, which was originally developed in UK. The ICECAP-SCM is a tool for measuring wellbeing in terms of ‘capability’ from various perspectives. The systematic and team-based approach in the translation process included the content validation of the tool. This study took into account the views of older people in the general population, nursing home residents, palliative care patients and palliative care professionals. In the future, this will allow cross-national comparisons of research results and the aggregation of data. The ICECAP-SCM can now be used and further tested for its psychometric properties in German-speaking countries for measuring the quality of life at the end of life, especially in palliative care. Providing valid measures for the assessment of quality of life at the end of life in multinational studies is a crucial step to improve palliative care in ageing societies.

## Supplementary Information

**Additional file 1.** ICECAP-SCM-English.pdf (Original questionnaire, English version).

## Data Availability

The datasets used and/or analysed during the current study are available from the corresponding author on reasonable request.

## Abbreviations

CVIs: Content Validity Indices
ICECAP-SCM: ICECAP Supportive Care Measure
ISAP: Institute of Social Medicine, Occupational Health and Public Health
I-CVIs: Item-specific Content Validity Indices
N: Sample size
S-CVIs: Scale Content Validity Indices
SD: Standard deviation
SPSS: Statistical Package for the Social Sciences
TRAPD: Translation, Review, Adjudication, Pretesting, and Documentation
