# Supplementary material for: Valuing end-of-life care: translation and content validation of the ICECAP-SCM measure
Source: BMC Palliat Care. 2021 Feb 8;20:29. doi: 10.1186/s12904-021-00722-5 (PMC7871540; doi:10.1186/s12904-021-00722-5)
Supplement: Supplementary file 1 — Additional file 1. ICECAP-SCM-English.pdf (Original questionnaire, English version). [file 12904_2021_722_MOESM1_ESM.pdf]

ABOUT YOUR WELL-BEING

Please place a tick (✓) in ONE box in EACH group below, to indicate which statement best describes your situation at the moment.

1) Having a say – Your ability to influence where you would like to live or be cared for, the kind of treatment you receive, the people who care for you

I am able to make decisions that I need to make about my life and care **most of the time**	4
I am able make decisions that I need to make about my life and care **some of the time**	3
I am able to make decisions that I need to make about my life and care **only a little of the time**	2
I am **never** able to make decisions that I need to make about my life and care	1

2) Being with people who care about you – Being with family, friends or caring professionals

If I want to, I am able to be with people who care about me **most of the time**	4
If I want to, I am able to be with people who care about me **some of the time**	3
If I want to, I am able to be with people who care about me **only a little of the time**	2
If I want to, I am **never able to** be with people who care about me	1

3) Physical suffering – Experiencing pain or physical discomfort which interferes with your daily activities

I **always** experience significant physical discomfort	1
I **often** experience significant physical discomfort	2
I **sometimes** experience significant physical discomfort	3
I **rarely** experience significant physical discomfort	4

4) Emotional suffering – Experiencing worry or distress, feeling like a burden

I **always** experience emotional suffering	1
I **often** experience emotional suffering	2
I **sometimes** experience emotional suffering	3
I **rarely** experience emotional suffering	4

ICECAP-SCM – individual v1.2 3rd February 2012© Joanna Coast, Eileen Sutton

Please place a tick (✓) in ONE box in EACH group below, to indicate which statement best describes your situation at the moment.

5) Dignity – Being treated with respect, being spoken to with respect, having your religious or spiritual beliefs respected, being able to be yourself, being clean, having privacy,

I am able to maintain my dignity and self-respect **most of the time**	4
I am able to maintain my dignity and self-respect **some of the time**	3
I am able to maintain my dignity and self-respect **only a little of the time**	2
I am **never** able to maintain my dignity and self-respect	1

6) Being supported – Having help and support

I am able to have the help and support that I need **most of the time**	4
I am able to have the help and support that I need **some of the time**	3
I am able to have the help and support that I need **only a little of the time**	2
I am **never** able to have the help and support that I need	1

7) Being prepared – Having financial affairs in order, having your funeral planned, saying goodbye to family and friends, resolving things that are important to you, having treatment preferences in writing or making a living will

I have had the opportunity to make **most** of the preparations I want to make	4
I have had the opportunity to make **some** of the preparations I want to make	3
I have had the opportunity to make a **few** of the preparations I want to make	2
I have **not** had the opportunity to make **any** of the preparations I want to make	1

Thank you for your help

ICECAP-SCM – individual v1.2 3rd February 2012© Joanna Coast, Eileen Sutton
